# Nutritional and Volatile Characterisation of Milk Inoculated with Thermo-Tolerant *Lactobacillus bulgaricus* through Adaptive Laboratory Evolution

**DOI:** 10.3390/foods10122944

**Published:** 2021-11-30

**Authors:** Jiahui Liang, Michelle Ji Yeon Yoo, Brent Seale, Gianpaolo Grazioli

**Affiliations:** 1 School of Science, Faculty of Health and Environment Sciences, Auckland University of Technology, Private Bag 92006, Auckland 1142, New Zealand; frg3269@aut.ac.nz (J.L.); brent.seale@aut.ac.nz (B.S.); 2 Giapo Research Kitchen, 12 Gore Street, Auckland CBD, Auckland 1010, New Zealand; gpg@giapo.com

**Keywords:** *Lactobacillus bulgaricus*, adaptive laboratory evolution, volatiles, amino acids

## Abstract

In this study, thermo-tolerant strain of *Lactobacillus bulgaricus* (*L. bulgaricus*) was developed using gradual increase in temperature to induce Adaptive Laboratory Evolution (ALE). Viable colony count of 1.87 ± 0.98 log cfu/mL was achieved at 52 °C, using MRS agar supplemented with 2% lactose. Changes in bacteria morphology were discovered, from rod (control) to filament (52 °C) to cocci after frozen storage (−80 °C). When milk was inoculated with thermo-tolerant *L. bulgaricus*, lactic acid production was absent, leaving pH at 6.84 ± 0.13. This has caused weakening of the protein network, resulting in high whey separation and lower water-holding capacity (37.1 ± 0.35%) compared to the control (98.10 ± 0.60%). Significantly higher proteolytic activity was observed through free amino acids analysis by LC-MS. Arginine and methionine (237.24 ± 5.94 and 98.83 ± 1.78 µg/100 g, respectively) were found to be 115- and 275-fold higher than the control, contributing to changing the aroma similar to cheese. Further volatile analysis through SPME-GC-MS has confirmed significant increase in cheese-aroma volatiles compared to the control, with increase in diacetyl formation. Further work on DNA profiling, metabolomics and peptidomics will help to answer mechanisms behind the observed changes made in the study.

## 1. Introduction

In yoghurt making, lactic acid bacteria (LAB) use the lactose in milk and produce lactic acid through fermentation. During the fermentation process, the pH is reduced from 6.8 to 4.5, resulting in the formation of milk gel network with changes in the casein structure. Factors such as type of LAB strain, incubation time and temperature during fermentation play a considerable role in stabilising the interactions of casein micelles and forming a three-dimensional gel network [1]. Exopolysaccharides (EPS) produced by the LAB during incubation bind with proteins creating different textures properties of yoghurt, including viscosity and thickness [2,3]. 

Some LAB strains used in yoghurt-making are considered probiotics, as they may help the gut microbiome and overall health. This has caused an increase in consumption and demand for probiotic dairy products in recent years [4]. The most common probiotic strains are the genera *Lactobacillus* and *Bifidobacterium* [4]. Different countries have different requirements in using LAB in yoghurt production. For example, in the U.S., the FDA requires dairy product fermentation to be done with two particular bacteria: *Streptococcus thermophilus* and *Lactobacillus delbrueckii* subsp. *bulgaricus* (*L. bulgaricus*) [4]. 

Proteolytic and lipolytic properties of LAB play an essential role in the degradation of proteins and lipids in milk. During proteolysis, enzyme breaks down the milk protein, releases free amino acids, and reduces sapid compounds (e.g., glutamic acid and citric acid) [5] with intracellular and extracellular enzymes. As lactate is the primary product of LAB fermentation, other flavour compounds such as diacetyl, acetoin, acetaldehyde, or acetic acid create specific flavour profiles for yoghurts produced from *Streptococcus thermophilus* and *L. bulgaricus*.

*L. bulgaricus* is an aerobic to anaerobic homofermentative bacteria that uses the Embden-Meyerhof-Parnas glycolytic pathway (EMP) [6]. In lactic acid fermentation, *L. bulgaricus* produces hydrogen peroxide through oxidation of reduced NADH to NAD^+^, resulting from the conversion of pyruvate into lactate. As a homofermentative lactic acid bacteria, it produces lactic acid as the primary by-product, along with proteolytic and diacetyl as well as organic acids and aromatic compounds [7]. 

Diacetyl is extensively used as food and beverage flavouring. Diacetyl formation in yoghurt is one of the most essential flavour contributors due to the low threshold for consumers to detect its presence. Diacetyl can be recognised by its distinctive buttery aroma in amounts as small as 1 mg/kg, in commercial yoghurt products, with a range from 0.2 to 3 mg/kg of diacetyl for a balanced buttery flavour in yoghurt [8,9] 

Recently, the use of different LAB has been trialed to accelerate the acidification, and to enhance the health benefits of yoghurt [10]. This approach has also resulted in favorable changes in nutritional and sensorial properties in yoghurt products to satisfy the demand over novel dairy products. 

Productivity and cost are the primary concern by the industry producing fermented foods. An increasing number of studies have demonstrated the selection of new LAB strains with different and specific functional properties, such as stress tolerance and production of molecules (enzymes, polysaccharides, aromatic compounds) [11,12]. 

Stress tolerance generally includes different kinds of variations during fermentation: temperature, pH, osmotic stress, and others. When fermentation is first started, the osmotic pressure changes due to the high concentration of solutes in the medium, which may lead to cell exposition causing water efflux from the cell, result in inactivation of the critical enzymes of microbial metabolism [13]. Later, during the fermentation process, pH and temperature change due to acid products and incubation temperatures. For yoghurt especially, the culture concentration and temperature have a significant effect on the physicochemical properties of the end products [14]. Based on the limitations on yoghurt production, obstacles such as maintaining its viability during the processing and storage, cost efficacy and resistance to the physicochemical processing must be considered [15]. These limitations can be improved by genetic engineering of the strains. However, careful consideration of using is required to develop strains that are suitable for the desired purpose, and safe for consumption. 

Various studies have used adaptive laboratory evolution (ALE) to control evolution and create strains with unique characteristics. ALE is a method to employ the fundamental mechanisms of molecular evolution and adaptive changes that are generated in microbial populations during long term selection under specific growth conditions [16]. ALE is believed to be methodologically straightforward to investigate the effect of evolutionary forces and develop unique strains with desirable phenotypes, performance, and stability [17]. In order to perform ALE on a micro-organism, a single strain culture is exposed to continuous propagation under stress in order for the organism to evolve, survive and grow under the conditions of the desired stress factor. This new phenotype is acquired through mutations in key metabolic genes [18] as well as genome and transcriptome reorganization [19]. 

Applications of ALE have been made to create new brewing yeast for ale and lager to improve the brewing efficiency and beer quality; *E. coli* for developing freeze-thaw stability for baker’s yeast and *Lactobacillus rhamnosus* through freeze-thaw cycles to select tolerant strains [20,21,22]. Applying ALE to *L. bulgaricus* in yoghurt production is a novel approach. The result of previous studies on ALE has given promising results in reducing obstacles and to maximise productivity and quality of different products. Therefore, ALE could be used to modify starter cultures to create yoghurt with unique nutritional, taste and aroma characteristics. 

Hence, the aim of this study was to apply ALE to *L. bulgaricus* in order to develop a thermo-tolerant strain using a gradual increase in growth temperature. The study also aimed to inoculate milk with the mutated thermo-tolerant strain and investigate subsequent changes observed in microbiological, physicochemical, and nutritional characteristics.

## 2. Materials and Methods

### 2.1. Isolation of L. bulgaricus

**Figure 1:**
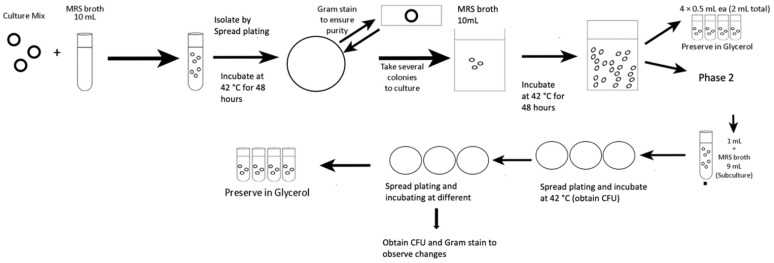
Flow diagram of isolation of *L. bulgaricus* pure culture at 42 °C and thermo-tolerant *L. bulgaricus* induction at 52 °C.

A commercial yoghurt starter, which consisted of *Streptococcus thermophilus* and *Lactobacillus delbrueckii* (Yoflex Express 1.1 powder, CHR Hansen, Hoersholm, Denmark) was used in the project. The starter culture of 0.1% (*w*/*v*) was added to 10 mL MRS broth made according to the manufacturer’s instruction (Difco Laboratories, Francisco Soria Melguizo, Madrid, Spain). Serial dilution was performed from 10^−1^ to 10^−7^ with MRS (De Man, Rogosa and Sharpe) broth. Each of the diluted samples was plated on MRS agar to calculate the colony forming unit (cfu/mL). *L. bulgaricus* was isolated and grown on elective media MRS agar (Difco Laboratories, Francisco Soria Melguizo, Madrid, Spain) with additional 2% of lactose as a source of energy (LMRS), which was sterilised at 121 °C for 15 min. *L. bulgaricus* plates were incubated anaerobically with BD GasPac Anaerobe Container System (Becton Dickinson Microbiology Systems) at 42 °C for 48 h in an incubator (Thermo Scientific™ Heracell™ VIOS CO_2_ incubator, Waltham, MA, USA). A colony was randomly selected from the plate, and morphology was observed by Gram staining. The process was repeated by sub-culturing the colony three times until the Gram staining appeared to be all Gram-positive and rod-shaped, to validate for collecting pure culture of *L. bulgaricus*. Once the culture was confirmed as pure *L. bulgaricus*, spread plating was performed in triplicate. The pure culture was then divided into 1 mL stock, containing 0.5 mL of pure culture in peptone water and 0.5 mL of 40% (*w*/*v*) glycerol/water, and stored at −80 °C for up to 3 weeks for characterisation of bacterial morphology with respect to change in growth temperature and freeze-thaw stability (Figure 1).

### 2.2. Application of Adaptive Laboratory Evolution to Generate Thermo-Tolerant Strain

The laboratory evolution experiments were adapted based on the protocol by Pena-Miller et al. (2013) [23]. ALE was applied to the purified *L. bulgaricus* by exposing them to a gradual change in growth temperature in repeated batch cultivations. The morphology changes of the strains were observed every 48 h. Using standard spread plate technique on LMRS agar plate, each of the purified sub-culture from Section 2.1 was firstly Gram-stained to check for morphology under a light microscope (Celestron Labs CB2000CF Compound Microscope, Torrance, CA, USA) at 1000× with paraffin oil. Then, the sub-culture was plated and incubated at 42 °C in anaerobic conditions in BD GasPac Anaerobe Container System (Becton Dickinson Microbiology Systems) for 48 h in an incubator (Thermo Scientific™ Heracell™ VIOS CO_2_ incubator). *L. bulgaricus* collected was harvested in peptone, and serially diluted with 0.1% of peptone water. Then, 0.1 mL of each serial dilution was inoculated by spread plating onto new LMRS plate, in triplicate. After 48 h, the culture was sub-cultured onto new LMRS plates in triplicate, and the growth temperature was increased by 1.0 °C, under the same incubation condition. The procedure was repeated and data were collected per 1.0 °C temperature increase until no viable counts were achieved. The viable counts were shown as means of the colony-forming unit log (CFU) g/mL. The subculture was then harvested, and the colonies from the plate were harvested to 1 mL of peptone water, then centrifugation at 3000 rpm for 5 min (Eppendorf Centrifuge 5430 R, Hamburg, Germany) at 25 °C. After removing the supernatant, the culture was preserved in glycerol, following the same protocol in Section 2.1, for further work on characterisation of bacterial morphology with respect to change in growth temperature and freeze-thaw stability (Figure 1). To ensure that the changes we were observing from the thermo-tolerant *L. bulgaricus* were consistent, at least five batches of *L. bulgaricus* have undergone ALE process (20 days per cycle) separately to develop thermo-tolerant *L. bulgaricus*, and survive to 52 °C, over 10 months of period.

#### Performance of Thermo-Tolerant *L. bulgaricus*

To measure the level of fitness of thermo-tolerant *L. bulgaricus*, amount of total lactic acid produced was quantified by using l-/d-Lactic acid assay (Megazyme International Ireland, Bray, Wicklow, Ireland) following the manufacturer’s instructions. A mixture of 1.60 mL of distilled water with solutions and suspension provided in the kit were used according to the manufacture’s guideline, with deionized water as blank [24]. Inoculated milk sample of 1 g and 60 mL of distilled water were added to a 100 mL volumetric flask. Into the dilution of the inoculated milk, 2 mL of Carrez I solution, 2 mL of Carrez II solution and 4 mL of NaOH solution were added and the volume was adjusted to 100 mL with distilled water. A 1.5 mL of this solution mixture was analysed according to the instruction of the kit [24]. As solution A1 was the mixture of samples and solution A2 was solution A1 with suspension 5 provide in the kit. Both d- and l-Lactic acid contents were measured for absorbance using a spectrophotometer (Spectrophotometer UV-VIS Model UV-1280, Shimadzu, Tokyo, Japan) at 340 nm. The equation below was used to calculate the concentration of the lactic acids. Absorbances of the solutions A1 was first absorbed after 3 min, and the absorbances of solution A2 was absorbed after 5 min of reaction time with A1. All of the samples were analysed in triplicate.

C [g/L] = (V × MW/ε × d × v) × ∆A(D/L)-lactic acid(1)

where: V = final volume (mL); MW = molecular weight of d-lactic acid or l-lactic acid(g/mol); ε = extinction coefficient of NADH at 340 nm, d = light path (cm); v = sample volume (mL); ∆A = the absorbance difference (A2 − A1).

### 2.3. Milk Inoculation and Incubation with L. bulgaricus

Milk was inoculated with *L. bulgaricus* that were isolated from Yoflex Express 1.1 powder used in Section 2.1. The control sample was prepared with *L. bulgaricus* grown at 42 °C (control strain) and the thermo-tolerant sample was prepared with thermo-tolerant *L. bulgaricus* grown at 52 °C. Starter culture was made by inoculating the pure stock culture (control, grown at 42 °C) or the thermo-tolerant culture (described in Section 2.2, grown at 52 °C) to 100 mL of Anchor™ Trim Milk of 0.1% fat sourced from a local supermarket to the final concentration of 0.01% (*w*/*v*). The inoculated milk was incubated in anaerobic condition with BD GasPac Anaerobe Container System (Becton Dickinson Microbiology Systems) and anaerobic chamber in a sealed and sterilised glass jar with a lid at the temperature of 42 °C for both control and thermo-tolerant cultures for 24 h, in an incubator (Thermo Scientific™ Heracell™ VIOS CO_2_ incubator). Samples were immediately transferred to a refrigerator and stored at 4 °C until further analysis. The described sample production was repeated three times. 

### 2.4. Inoculated Milk Analyses

#### 2.4.1. Determination of pH and Viscosity

A pH meter with a glass electrode (Thermo Scientific Orion Star A211e, Waltham, MA, USA) was calibrated and then measurements were taken from 10 g of inoculated milk samples. Brookfield viscometer (RST-CC Coaxial Cylinder, Middleborough, MA, USA) with Bob 40 mm Brookfield Ametek spindle (CCT-40, Middleborough, MA, USA.) measuring system was used to measure the viscosity. Both control and thermo-tolerant samples were tested in triplicates. Each sample was filled up to the maximum level marked in the sample holder (approximately 68 mL). The viscometer was then operated at a speed of 400 rotations per second for 2 min. Rheo3000 programmed to the viscometer was used to control the shear stress or shear rate, and to calculate yield and average viscosity. All viscosity measurements for each sample were performed in triplicate.

#### 2.4.2. Water Holding Capacity (WHC)

Method used for determining the water holding capacity (WHC) was modified from [25]. Inoculated milk sample of 10 g was placed in a 15 mL centrifuge tube and centrifuged at 3000 rpm for 10 min at 4 °C. The supernatant was collected and weighed. Measurements were taken in triplicate. The following equation were used to determent the WHC value:

WHC (%) = (weight of 10 g of inoculated milk sample − weight of supernatant)/weight of 10 g of inoculated milk sample × 100(2)

#### 2.4.3. Determination of Free Amino Acids with Liquid Chromatography-Mass Spectrometry (LCMS)

A 40-µL sample of supernatant of inoculated milk was placed in a 1.5 mL polypropylene microcentrifuge tube, mixed with 40 µL of methanol containing 10 mg/L of d4-alanine as internal standard-spiked methanol. LC-MS (Agilent 1260 Infinity Quaternary LC System, Santa Clara, CA, USA) equipped with Kinetex Evo C18 (150 mm × 2.1 mm × 1.7 µm. Phenomenex, Torrance, CA, USA) column was used. Preparation and analysis methods used by the same research group, [26] were followed. Each of the inoculated milk sample was analysed three times.

#### 2.4.4. Determination of Volatile Compounds with Solid Phase Microextraction—Gas Chromatography-Mass Spectrometry (SPME-GC-MS)

Volatile components of the inoculated milk samples were analysed according to the methods used by the same research group [27] with slight modifications. 

A sample of approximately 1 g of inoculated milk sample was quickly introduced to 10 mL headspace vial, followed by addition of 4 µL of internal standard (2-chloro-phenol). It was then sealed with a polytetrafluoroethylene silicon septum. The vials were then incubated at 50 °C in a thermal block for 15 min with agitation. The Solid Phase Microextraction (SPME) fibre of 50/30 µm Divinylbenzene/Carboxen/Polydimethylsiloxane (DVB/CAR/PDMS), StableFlex fiber 24 ga, with length of 10 mm (Supelco, Bellefonte, PA, USA) was exposed to the sample headspace for 5 min. The volatile compounds were separated by Agilent DB-FATWAX UI column (30 m × 0.25 mm × 0.25 µm, Santa Clara, CA, USA) with helium as a carrier gas at a constant flow rate of 1.1 mL/min, using Gas Chromatography-Mass Spectrometry (GC-MS) (Agilent Technologies 7890B GC-System & 5977B MSD, Santa Clara, CA, USA). 

The mode of injection was splitless, and the inlet temperature for the injection port was set to 250 °C with 45 mL/min split flow and 2 min splitless time. Chromatographic conditions were as follows: the oven was held at 40 °C for 2 min, then raised to 240 °C at a rate of 8 °C/min and held for 10 min. The total run time was 37 min. The MS was operated in the electron impact mode (EI) with a source temperature of 230 °C, a quadrupole temperature of 150 °C, an ionising voltage of 70 eV, and transfer line temperature of 250 °C. The mass spectrometer scanned masses from 38 to 450 m/z.

Samples were analysed in at least triplicate. The concentration of volatile compounds in the inoculated milk samples were calculated using the following formular:

[P] = (AP/AIS) × [IS](3)

where: P (µg/L) = the concentration of the product; IS (µg/L) = the concentration of internal standard; AP = the area of the product; AIS = the area of the internal standard

#### 2.4.5. Determination of Odour Threshold and Relative Odour Activity Value (OAV) 

Odour threshold values (OAV) published by Leffingwell & Associates (2020) [28] were used to calculate the OAV of inoculated milk samples. Relative OAV for each volatile compound was calculated using OAV = c/t, where c is the total compound concentration in the inoculated milk, and t is the odour threshold value. For the compounds with a range of odour thresholds rather than a single number (e.g., 1-Octanol, odour threshold 110–130), the lowest value was used to calculate the relative OAV. Only the compounds with relative OAVs greater than 1 were counted to contribute to the inoculated milk aroma. Odour descriptions of volatiles were adapted from [29,30].

### 2.5. Statistical Analysis

Data were analysed using Rstudio (Version 1.3.1093, Boston, MA, USA) and presented as mean ± standard deviation. All tests were carried out in triplicate and analyzed by One-Way Analysis of Variance (ANOVA). Fisher’s (LSD) multiple comparison tests were used to differentiate the differences between the means. A *p*-value < 0.05 was considered statistically significant. 

## 3. Result and Discussion 

### 3.1. Generation of Thermo-Tolerant Strains of L. bulgaricus

**Figure 2:**
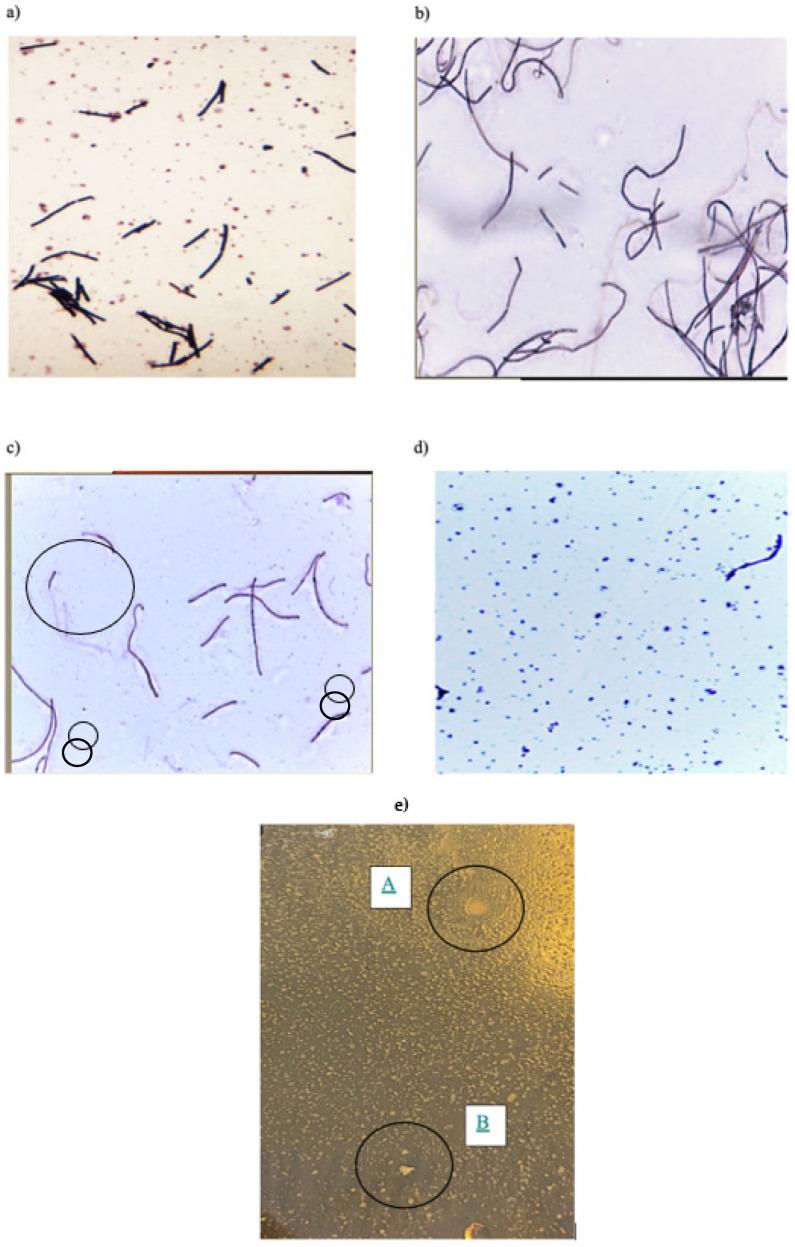
Microscope images (×1000 magnification) of Gram-stained *L. bulgaricus*. (**a**) Pure *L. bulgaricus* strain incubated at 42 °C, showing short and rod shape. (**b**) Thermo-tolerant strain of *L. bulgaricus* incubated at 52 °C, showing elongated shape. (**c**) First incubation at 52 °C after storing at −80 °C for 3 weeks, showing truncation in elongated cells and formation of cocci. (**d**) Post to three rounds of sub-culturing at 52 °C, showing further transformation of truncated elongated rod-shaped cells (from (**c**)) into cocci. (**e**) Colony A and B are examples of *L. bulgaricus* showing irregular edges at 52 °C.

Starting from the isolated pure culture of *L. bulgaricus* grown at 42 °C, temperature elevation was used as an environmental stimulus to induce ALE. Over the course of 20 days, sub-culturing of bacteria, which survived the elevation of temperature, was performed, and at every temperature point, the bacterial cells were examined for morphology under a light microscope. As shown in Figure 2a, initially, the control, *L. bulgaricus* grown at 42 °C had short, rod-shaped cells, with 7.62 log CFU/mL on average when grown on LMRS. We were able to increase the temperature to 52 °C and achieve 4.66 log CFU/mL. The survival rate of *L. bulgaricus* is known to decrease due to the loss of its polysaccharide production ability starting from 47 °C. Hence supplementation with lactose at 2% to MRS has helped with development of *thermo-tolerant strain*. Studies have shown that adding lactose has increased the growth of *L. bulgaricus* and provided effective protection to *L. bulgaricus* in extreme temperature [31,32]. Lactose can replace structural water molecules in membranes after dehydration at extreme temperature, preventing protein unfolding and aggregation by hydrogen bonding with protein polar groups [33]. As shown in Figure 2b, the thermo-tolerant strain showed change in bacterial morphology to elongated cells compared to the short rod shapes at 42 °C, as a response to environmental stimuli, as explained by [34]. The enhanced capability of bacteria to survive after exposure to a lethal temperature has been associated with the production of heat-shock proteins [35,36]. Filamenting temperature-sensitive mutant Z (FtsZ) is the first tubulin-like protein to respond during cell division. FtsZ recruits other synthesis proteins that produce Z-ring for cell division and form filaments with smooth morphology as elongation [37]. As the colony count observed in this study was much less than expected, it was assumed that the thermo-tolerant bacteria is lacking FtsZ, resulted in no cell divison but elongation into filaments [38]. 

When both the control and the thermo-tolerant strains were frozen at −80 °C in glycerol for three weeks to test for freeze-thaw stability, another change in morphology was detected but only in the thermo-tolerant strain. This strain was re-incubated and sub-cultured on LMRS agar for 48 h. As shown in Figure 2c, morphology has changed to shorter chins and cocci on the first sub-culture. After three rounds of sub-culturing (48 h per sub-culture), more of the rod-shaped cells were converted into cocci (Figure 2d), with irregular edges (circled in Figure 2e). The observations may be explained as adaptation mechanism of *L. bulgaricus* to the heat stress by taking appropriate molecular responses to reduce the extreme effects and restore and survive [39]. The observed change in morphology may be caused by mutation occurring in MreB, which is an actin-like cytoskeletal protein found in most rod-shaped bacteria. Previously, transition from growing as a rod-shaped cell to coccus has been observed in *E. coli*, *Arthrobacter*, *Acinetobacter*, and *Rhodococcus equi* [40]. 

There is a possibility that new microbial communities comprised of a mixture of rod and coccus shapes had formed in a layered structure, with a group of cocci layering atop of the rod shape cells, as proposed by [41]. In the current study, *L. bulgaricus* was sub-cultured on a new LMRS plate every 48 h. The nutrient was delivered from the bottom of the plate which would favour the rod-shapes cells, in the layered structure. This would have compromised the fitness level of cocci at the expense of the rod-shaped cells. With the favourable position in the biofilm, *L. bulgaricus* strains showed stronger rod-shaped cells at the bottom, small cocci at the top, forming white larger colonies with rough edges. Similar observations have also been reported in multispecies biofilms [42,43]. 

Supplementation of lactose to the growth medium (LMRS) has provided protective effect during the freezing storage of *L. bulgaricus*, as proposed by [36]. Sugars are able to replace structural water molecules in membranes after dehydration to prevent unfolding and aggregation of proteins by hydrogen bonding with polar groups of proteins [33]

### 3.2. Level of Fitness for Thermo-Tolerant L. bulgaricus and pH

*Lactobacillus* feed on lactose during fermentation and produce lactic acid, which is the major contributor to flavour and acidity of yoghurt. About 20 to 40% of lactose is transformed into lactic acid during fermentation, resulting in around 0.9% of the lactic acid [29]. In the current study, L-lactic acid and D-lactic acid produced by the thermo-tolerant strain of *L. bulgaricus* were compared against the control (grown at 42 °C) to determine whether the mutation has caused a decline in its primary role of producing lactic acid or not. Measurements of lactic acid was taken from inoculated milk samples that were produced by inoculating with the two bacterial strains: thermo-tolerant and wild-type control. In the inoculated milk produced from the control strain, 7.62 ± 0.23 log CFU/mL from LMRS was inoculated, and 7.41 ± 0.36 log CFU/mL was observed post fermentation. However, with the thermo-tolerant mutant strain, a large drop in viable counts was observed between inoculated and post fermentation of 4.66 ± 0.59 and 1.87 ± 0.98 log CFU/mL, respectively. This result indicated that the weaker thermo-tolerant *L. bulgaricus* mutant loses viability during the fermentation at higher temperatures.

L-lactic acid is the predominant metabolite formed during fermentation of milk. D-lactic is an isomer, found in small amount in yoghurt that causes acidosis [44]. Both L- and D- lactic acid isomers were found in the control inoculated milk samples; 5.40 ± 0.20 and 2.13 ± 0.80 g/L, respectively, where neither of the lactic acid isomers were found from milk inoculated with the thermo-tolerant strain. The result may be explained by the lack of lactate dehydrogenase, which is the primary enzyme for lactic acid production. A decrease in interconversion of pyruvate and lactate using NADH and NAD^+^ may have taken place, as proposed by [45]. Another possible explanation could be that by-product (ethanol or acetic acid) formation may have interfered with the lactic acid production [46]. 

There was a significant (*p* < 0.05) difference in pH between the inoculated milk samples made from the two different strains. Samples made from the thermo-tolerant strain had average pH of 6.84 ± 0.13, which was much higher than the average pH of yoghurt in general, 4.2 to 4.6, and compared to the control in the study, 4.55 ± 0.04. Low pH in inoculated milk results from precipitation of casein and their coagulation, producing dense texture. With the absence of lactic acid in inoculated milk made with the thermo-tolerant strain, low level of casein cross-linking would have taken place to form the gel network. Although Lucey et al. (2001) [47] claims that the use of a high incubation temperature for yoghurt production increases the rate of acidification, the concept was not applicable to having bacteria grown in higher temperature as a starter culture. Despite the higher pH, the texture of the inoculated milk after fermentation remained firm, and this could be explained by the production of EPS from the bacteria. Further testing of the production of EPS will be required to verify this explanation.

### 3.3. Physicochemical Properties 

#### 3.3.1. Viscosity

**Figure 3:**
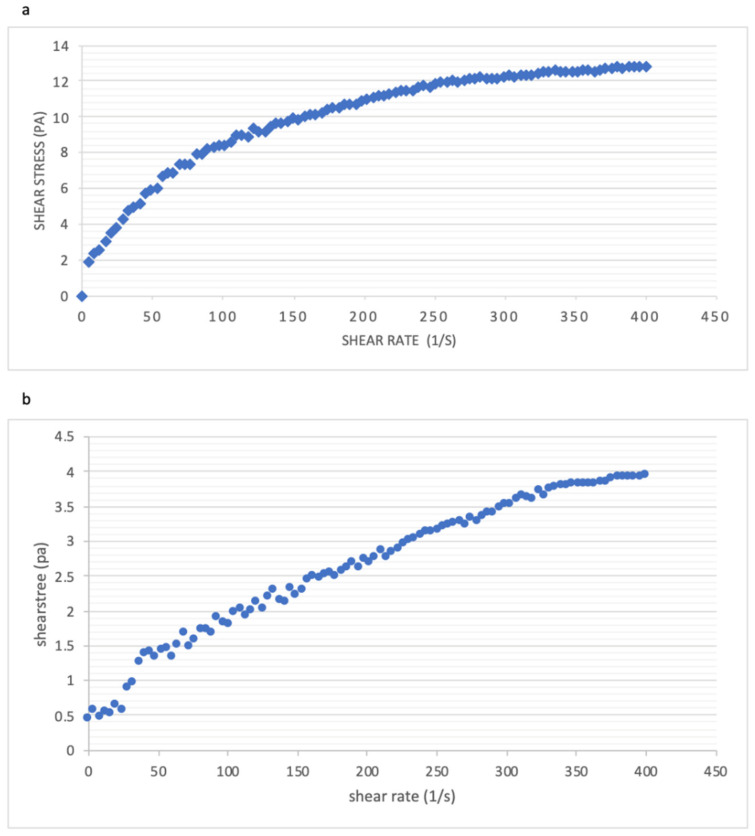
Shear stress against shear rate measured in milk inoculated with: (**a**) the control strain (42 °C) and (**b**) the thermo-tolerant strain (52 °C).

Yield stress is used to determine the extent of cross-linking in yoghurt, and this is translated as the firmness of yoghurt [48]. Usually the use of a single strain of *Lactobacilli* is known to produce the lowest firmness and curd tension in yoghurt production [49]. Both of the inoculated milk samples produced from the control and the thermo-tolerant strains were incubated at 42 °C for 24 h for stable texture and viscosity and to minimise syneresis. As shown in Figure 3, both samples have shown a typical trend of shear thinning and yield stress behaviour, as reported by Bulter and McNulty (1995) [50]. The control sample required approximate 2 Pa of yield stress applied before the flow. The entangled molecules in the milk gel structure have started to untangle from stress rate of 0 to 72.72 s^−1^ and became less resistant to flow, clearly demonstrating a shear-thinning behaviour (Figure 3a). The control sample had higher yield stress compared to that of the thermo-tolerant strain and this has prevented syneresis, whey separation and breaking down in structural alignment, as described by Saleh et al. (2020) [33]. Samples made from thermo-tolerant strain showed a lower yield stress, 0.5 Pa. Compared to the control sample, the flow curve appeared to be more linear. The observations over the shear-thinning behaviour may be explained by weakened alignment of the biopolymer-biopolymer molecule interactions or biopolymer molecules with the shear field [48,51] and from the aggregation of casein particles influencing the rheological behaviour of inoculated milk overall [47,52]. The shear-thinning behavior can further be linked to the WHC data in the next section. 

#### 3.3.2. WHC

Inoculated milk samples produced from the control strain showed more than a double in WHC when compared to the thermo-tolerant strain; 98.10 ± 0.60 and 37.1 ± 0.35%, respectively. The variation may be explained by possible rearrangements of casein and their cross-links within the inoculated milk gel network, causing formation of large pores in the samples made from the thermo-tolerant strain [47], and also the weakening of the gel network from the lack of pH decline during the fermentation process. These may have caused faster and more severe syneresis in the inoculated milk produced from the thermo-tolerant strain.

#### 3.3.3. Free Amino Acid Profile 

A total of 27 FAAs was identified in the inoculated milk samples, with total FAAs content of 225.25 ± 37.45 and 1747.42 ± 34.69 μg/100 g, for the control and the thermo-tolerant strain, respectively. As shown in Table 1, most of the individual FAAs detected in the current study were significantly different between those made from the control and the thermo-tolerant strain (*p* < 0.001). Significantly higher proteolytic activity, measured from the release of FAAs [53], was found in inoculated milk produced from the thermo-tolerant strain in both total FAAs amount and also in individual FAAs. Approximately 7.76 times higher in total FAAs was found when compared to the use of control. There were number of FAAs which differed in great quantity between the two samples. Glutamic acid was found 4.8 times higher than the control sample; 265.88 ± 5.08 and 55.27 ± 9.67 µg/100 g, respectively. Glutamate dehydrogenase (GDH) is known to use NADH or NADPH as coenzymes to produce glutamic acid from 2-oxoglutaric acid in the tricarboxylic acid cycle [54]. Mutation may have occurred through the generation of thermo-tolerant strain by ALE in the current study, and this may have caused higher activity of the GDH. As shown in Table 1 the concentration of the following amino acid found in milk inoculated with the thermo-tolerant strain increased greatly compared to the control sample: histidine (19.95 times), arginine (115.73 times), threonine (10.31 times), ornithine (55.64 times), methionine (274.52 times), valine (19.27 times), lysine (7.69 times), tyrosine (37.27 times), isoleucine (14.99 times), leucine (47.67 times), phenylalanine (22.56 times) and tryptophan (19.63 times). The observed increases in FAAs may have been caused by the release of the hydrolysed polypeptide chain from storage proteins by exopeptidases [55]. It is likely that the exopeptidases have hydrolysed peptide bonds in the inoculated milk and continued the hydrolysis to a higher degree compared to the control sample [56,57]. Arginine and methionine have increased over 100 folds or more compared to the control. The increase in arginine may be explained by the high yield of glutamic acid [58] and the pH being high in the inoculated milk produced by the thermo-tolerant strain, which favours arginine production [59]. Arginine and methionine are classified as functional amino acids that helps with immune defense and as antioxidant [60]. One of the primary roles of *L. bulgaricus* is to hydrolyse milk protein, casein, which degrade into peptides and FAAs. Some of these FAAs also contribute to odour as precursors for volatile compounds as shown in Table 2 and Table 3 [61,62,63]. With the mutation occurring in the thermo-tolerant strain, the proteolytic activity of *L. bulgaricus* has improved. Further analysis using metabolomics and peptidomics may help to answer the change in metabolic pathways and the resultant amino acid profile. 

#### 3.3.4. Volatile Compounds 

A total of 56 volatile compounds were detected by SPME-GC-MS in both inoculated milk samples, which exceeded the findings of FAAs in other yoghurt studies reported in the literature. With the use of *Streptococcus thermophilus* as a single starter strain in yoghurt fermentation, 53 volatile compounds have been reported [64], where 31 volatile compounds were attributed to the use of cow milk [65]. The 56 volatile compounds detected in this study were sub-classified into chemical groups as shown in Table 2, as 10 carboxylic acids, 8 aldehydes, 11 ketones, 17 alcohols and 10 esters. Although many volatile compounds were detected, not all of them contribute significantly to the overall flavour and aroma profile of yoghurt. According to the literature, the major volatile compounds found in yoghurt are acetaldehyde, ethanol, acetone, diacetyl, and 2-butanone, and these are known to have a significant impact on the flavour [29,66,67]. However, acetaldehyde was not detected from neither of the inoculated milk samples tested in this study. Similar finding was reported by Raya et al. (1986), suggesting that acetaldehyde had not formed from pyruvate due to the absence of α-carboxylase or aldehyde dehydrogenase which generate acetaldehyde in *L. bulgaricus* [68]. However, acetic acid was found in both samples. A large difference between the control and the thermo-tolerant strain inoculated milk was found in hexanoic acid, octanoic acid and benzoic acid, where a double or more of the concentration was found in the control. Except for oxalic acid and 2-methyl-propanoic acid, the other carboxylic acids detected in milk inoculated with the thermo-tolerant strain were much lower in concentration compared to the control. 3-methyl-butanoic acid was found in both of the samples, which must have been converted from 3-Methylbutanal through leucine catabolism, as shown in many dairy products, contributing to the pleasant fresh cheese aroma [69,70]. 

A high amount of diacetyl (2,3-butanediol) production was evident in both samples made from the control and the thermo-tolerant strain, 313.62 ± 0.20 and 844.79 ± 0.13 µg/L, respectively. This compound is known to carry buttery and cheese-like odour, which was found to be approximately 2.7 times higher than the control, shifting the overall volatile profile to be more on cheese-like side. This may be partly explained by the use of growth medium supplemented with lactose in the current study. Growth medium used for LAB has been reported to play a significant role in diacetyl production. According to Choi et al. (2016) [71], MRS media enriched with glucose and citrate supplement increased more than 3 fold in diacetyl production compared to the minimal media. We have attempted to grow *L. bulgaricus* in both MRS media only and MRS supplemented with lactose. From our preliminary experiments, survival rate of *L. bulgaricus* was much higher with the increase in temperature through ALE with supplementation of lactose, as additional nutrient. Similar finding has been reported in *L. lactis* grown in modified culture mediums (potassium phosphate buffer with 0.5% glucose; reconstituted skim milk with 1% glucose), which over-expressed NADH oxidase to re-route the metabolic flux away from lactate production against oxidised product in metabolic pathway of citrate, resulting in increased diacetyl production to 0.36 to 0.38 g/L [72,73].

Among the 11 ketone compounds detected, the key contributors to the overall flavor profile were 2,3-butanedione (313.62 ± 0.20 and 844.79 ± 0.13 µg/L), 2-heptanone (126.81 ± 0.04 and 2045.73 ± 0.35 µg/L) and 2-nonanone (50.58 ± 0.27 and 12.50 ± 0.77 µg/L), in milk inoculated with the control and the thermo-tolerant strain, respectively. Ester biosynthesis by lipase and LAB is favoured by alcoholysis in fermented dairy products [74]. Alcoholysis is a trans-esterification reaction, which uses alcohol and fatty acyl-CoAs derived from the metabolism of fatty acids, amino acids, or carbohydrates in milk [75] to produce new ester and new alcohol compounds. As shown in Table 2, there was an increased activity of esterification and alcoholysis in the samples inoculated with the thermo-tolerant *L. bulgaricus*, where a huge increase in concentrations were observed when compared to the control in ethyl butyrate, ethyl hexanoate, ethyl octanoate and ethyl decanoate. Volatile compounds such as ethyl butanoate and ethyl decanoate are common esters found in cheese. Overall, the presence of these compounds carrying cheese aroma have contributed to development of off-flavour in the inoculated milk produced from the thermo-tolerant strain. There was an excessive amount of alcohols in general, in milk inoculated with the thermo-tolerant strain compared to the control. We hypothesise that the excessive production of alcohols as by-products may have interfered with production of lactic acid by the thermo-tolerant *L. bulgaricus*, and alcoholysis may be the key driver in over-production of alcohols in the thermo-tolerant strain. 

#### 3.3.5. Odour Threshold and Relative OAV 

OAV is a ratio of the odour threshold concentration to the volatile compounds, indicating how the volatile profile of the measured sample is contributed by each compound. The greater the OAV value, the greater the overall odour. Due to the limited information on the OAV in similar studies, only the dominating odour/flavour profiles of the inoculated milk samples were compiled to Table 3. Values greater than their threshold concentrations (relative OAVs > 1) were identified as odour-active compounds. In the control sample, four carboxylic acids have shown relative OAVs of greater than 1, with butanoic acid being the dominating compound (OAV of 19.47). For aldehydes, 2-Nonenal (E) was the highest with OAV of 632, followed by nonanal, heptanal and 4-Heptenal, (Z). For ketones, 2-nonanone was the most dominant (OAV of 19.65) and for alcohols and esters, 1-octen-3-ol (OAV 45.45) and ethyl butyrate (OAV 51.60) were the dominating compounds. Overall, the odour compound of the control samples appeared to be fruity with a mushroom note. 

In contrast, the relative OAVs of volatile compounds found in milk inoculated with the thermo-tolerant strain had some similarity compared to that of the control. Butanoic acid was the dominating compound for carboxylic acid group, with similar OAV value, of 14.69. 2-nonenal (E) was again the dominating compound for aldehydes (OAV 156.25), but in much smaller value compared to that of the control. There were three dominant ketone compounds with 2-nonanone being the highest (OAV 376.31), four dominating compounds of alcohols and two esters with 2-heptanol (OAV 139.98) and ethyl butyrate (OAV 607.84) being the most significant. The overall description of the milk inoculated with the thermo-tolerant strain was more on the cheesy and oily with pineapple notes. 

Significant decreases in ethyl butyrate (by 7.7 times), heptanal (by 5.72 times), 2-nonenal (E) (by 4.05 times) and 4-heptenal (Z) (by 6.23 times) and significant increases in ethyl hexanoate (by 39.5 times) and 2-nonanone (by 19.15 times) were found in the OAVs, comparing the milk inoculated with the control to the thermo-tolerant strain. An alcohol compound, 2-heptanol, was absent in the control but found in high amount in the milk inoculated with the thermo-tolerant strain. As shown in Table 3, it was evident that these volatile compounds have contributed significantly in changing the overall odour of the inoculated milk from the control. Contribution of cheese-like flavour from the increase in FAAs, such as methionine, phenylalanine, threonine and branched chain amino acids (leucine, isoleucine and valine) [62] is also noteworthy (Table 1).

## 4. Conclusions

Thermo-tolerant *L. bulgaricus* was developed through spontaneous mutation by the application of ALE for the first time. Over the repeated exposure to elevated growth temperature over time, the thermo-tolerant strain survived to 52 °C albeit a clear change in morphology and reduced production of lactic acid during fermentation. Molecular mechanisms behind these observations remain to be fully understood and require further investigation. Milk inoculated with the thermo-tolerant *L. bulgaricus* has higher proteolytic activity, producing more free amino acids, particularly essential amino acids, when compared to the control. Significantly increased production of volatile compounds was also found in the milk inoculated with the thermo-tolerant *L. bulgaricus* and these have contributed to a cheese-like aroma. Stress caused by exposure to elevated growth temperature may have caused incomplete or modified metabolism in *L. bulgaricus* which may have caused production of organic acids. Competing micro-organisms in the milk due to the lack of acidification could also have contributed to these metabolites being produced. Increase in diacetyl formation was also evident, contributing to change in volatile profile to cheese-like aroma, compared to the control. Further work on DNA profiling, metabolomics and peptidomics will help to answer mechanisms behind the observed changes made in the study. Measurements in EPS, antioxidant and ACE-inhibitory activities and use of better imaging microscopy (e.g., FE-SEM) will help to charaterise the behavior of the thermo-tolerant *L. bulgaricus*.

## Figures and Tables

**Table 1:** FAA profile of inoculated milk made from the control and thermo-tolerant strain of *L. bulgaricus* using LC-MS. Data are presented as means ± SD, from the highest to the lowest quantity. Letters ^a^ and ^b^ indicate statistical difference (*p* < 0.001) across each row.

	Control (μg/100 g)	Thermo-Tolerant (μg/100 g)
Essential amino acid		
Lysine	22.73 ± 3.22 ^a^	174.79 ± 1.90 ^b^
Phenylalanine	6.09 ± 1.05 ^a^	137.45 ± 2.25 ^b^
Threonine	4.80 ± 0.78 ^a^	49.49 ± 1.17 ^b^
Histidine	4.63 ± 0.23 ^a^	92.36 ± 2.4 ^b^
Valine	4.62 ± 0.87 ^a^	89.02 ± 2.05 ^b^
Leucine	4.59 ± 0.77 ^a^	218.79 ± 4.36 ^b^
Isoleucine	2.78 ± 0.46 ^a^	41.66 ± 1.12 ^b^
Tryptophan	1.20 ± 0.20 ^a^	23.55 ± 0.31 ^b^
Methionine	0.36 ± 0.07 ^a^	98.83 ± 1.78 ^b^
Non-essential amino acid		
Glutamic acid	55.27 ± 9.67 ^a^	265.88 ± 5.08 ^b^
Proline	40.76 ± 7.19 ^a^	17.52 ± 0.42 ^a^
Ethanolamine	22.72 ± 3.93 ^a^	4.07 ± 0.14 ^a^
Alanine	22.29 ± 4.02 ^a^	79.98 ± 2.63 ^b^
Glycine	9.57 ± 1.58 ^a^	14.31 ± 0.34 ^a^
Taurine	8.34 ± 1.44 ^a^	6.52 ± 0.26 ^a^
Serine	2.65 ± 0.32 ^a^	0.28 ± 0.04 ^b^
Aspartic acid	2.59 ± 0.47 ^a^	1.11 ± 0.05 ^a^
Tyrosine	2.35 ± 0.35 ^a^	87.59 ± 2.89 ^b^
Arginine	2.05 ± 0.35 ^a^	237.24 ± 5.94 ^b^
Ornithine	1.86 ± 0.16 ^a^	103.49 ± 2.09 ^b^
Citrulline	1.03 ± 0.10 ^a^	2.30 ± 0.04 ^b^
Alanine	0.59 ± 0.09 ^a^	0.19 ± 2.63 ^a^
L_α_Amino-n-butyric acid	0.57 ± 0.11 ^a^	0.26 ± 0.02 ^a^
δ_Hydroxylysine	0.31 ± 0.08 ^a^	0 ^a^
Hydroxy-L-proline	0.19 ± 0.03 ^a^	0.18 ± 0.01 ^a^
Γ-amino-n-butyric acid	0.19 ± 0.03 ^a^	0.42 ± 0.02 ^a^
Sarcosine	0.07 ± 0.01 ^a^	0.04 ± 0.01 ^a^
DL-β-aminoisobutryic acid	0.03 ± 0.01 ^a^	0.11 ± 0.01 ^b^
Total free amino acids	225.25 ± 37.45	1747.42 ± 34.69

**Table 2:** Volatile compounds in inoculated milk samples analysed by SPME-GC-MS are shown. Data are presented as mean ± standard deviation (*n* = 3). M/z refers to mass-to-charge ratio, RT refers to retention time, RI refers to retention index and ND refers to not detected.

Compound	m/z	RT (min)	RI	Concentration (µg/L)	
				Control	Thermo-Tolerant
Carboxylic acid					
Oxalic acid	89.90	3.17	929.00	158.34 ± 0.19	7973.73 ± 3.45
Acetic acid	60.02	11.75	1443.27	1619.33 ± 1.12	1198.05 ± 2.13
Propanoic acid, 2-methyl-	88.05	13.66	1563.69	14.06 ± 0.33	130.81 ± 0.34
Butanoic acid	88.05	14.52	1620.85	4675.15 ± 3.76	3523.13 ± 1.21
Pentanoic acid	102.07	16.18	1735.18	30.38 ± 0.61	18.97 ± 0.31
Hexanoic acid	116.08	17.60	1838.63	9195.56 ± 4.32	5026.78 ± 0.73
Octanoic acid	144.12	20.35	2053.31	4251.74 ± 1.11	2209.72 ± 2.13
Nonanoic acid	158.13	21.68	2164.51	0.76 ± 0.00	9.71 ± 0.20
Benzoic acid	122.04	24.62	2430.28	2304.57 ± 0.52	822.18 ± 0.78
Dodecanoic acid	200.18	25.14	2479.94	118.84 ± 0.94	177.05 ± 0.64
Aldehyde					
Pentanal	86.07	3.68	969.00	249.09 ± 0.83	69.44 ± 0.63
Hexanal	100.09	5.30	1073.77	107.97 ± 0.74	ND
Heptanal	114.10	7.13	1177.34	152.60 ± 0.37	26.64 ± 0.32
4-Heptenal, (Z)-	112.09	8.17	1235.22	12.37 ± 0.27	154.09 ± 0.62
Nonanal	142.14	10.87	1390.06	57.98 ± 0.08	33.29 ± 0.34
Methional	104.03	11.82	1447.77	12.02 ± 0,61	10.79 ± 0.12
Benzaldehyde	106.04	12.92	1515.97	602.83 ± 0.26	195.73 ± 0.12
2-Nonenal, (E)-	104.12	13.19	1533.35	50.58 ± 0.27	12.50 ± 0.77
Ketone					
Butanone	72.06	2.73	891.00	170.66 ± 0.51	459.19 ± 0.40
2,3-Butanedione	86.04	3.65	967.00	313.62 ± 0.20	844.79 ± 0.13
2-Pentanone	86.07	3.66	968.16	313.62 ± 0.63	845.02 ± 0.13
2,3-Pentanedione	100.05	4.92	1051.51	75.49 ± 0.12	8.27 ± 0.32
3-Heptanone	114.10	6.55	1145.18	28.69 ± 0.01	9.11 ± 0.01
2-Heptanone	114.10	7.10	1175.84	126.81 ± 0.04	2045.73 ± 0.35
2-Nonanone	142.14	10.80	1385.67	98.23 ± 0.51	1881.56 ± 0.87
3-Octen-2-one	126.10	11.11	1404.45	6.09 ± 0.12	ND
1-Propanone, 1-(2-furanyl)-	124.05	13.71	1567.32	59.12 ± 0.68	18.65 ± 0.76
2-Undecanone	107.17	14.17	1597.26	54.96 ± 0.32	222.63 ± 0.49
2-Tridecanone	198.20	17.20	1809.13	12.67 ± 0.12	49.41 ± 0.77
Alcohol					
2-Butanol	74.07	4.48	1024.77	2.34 ± 0.15	16.94 ± 0.64
1-Propanol	60.06	4.69	1037.41	2.48 ± 0.01	61.96 ± 0.70
1-Butanol	74.07	6.69	1153.23	7.27 ± 0.34	33.59 ± 0.41
1-Pentanol	88.09	8.45	1250.99	380.14 ± 0.91	127.93 ± 0.18
2-Heptanol	116.12	9.69	1320.80	ND	419.95 ± 0.02
1-Hexanol	102.10	10.24	1353.39	159.70 ± 0.73	181.99 ± 0.91
1-Octen-3-ol	128.12	11.84	1448.93	45.45 ± 0.82	19.08 ± 0. 19
1-Heptanol	116.12	11.94	1455.01	117.50 ± 0.38	58.30 ± 0.23
1-Hexanol, 2-ethyl-	130.14	12.49	1488.77	754.34 ± 0.94	1945.54 ± 0.76
2-Nonanol	144.15	12.97	1519.23	ND	189.36 ± 0.83
2,3-Butanediol	90.07	13.25	1537.43	24.49 ± 0.42	6641.79 ± 0.18
Linalool	154.14	13.37	1545.42	46.18 ± 0.85	10.41 ± 0.62
1-Octanol	130.14	13.56	1557.23	31.98 ± 0.76	158.46 ± 0.10
2-Undecanol	172.18	15.95	1718.99	21.66 ± 0.37	8.74 ± 0.99
1-Decanol	158.17	16.54	1761.46	6.05 ± 0.17	109.51 ± 0.57
Benzyl alcohol	108.06	18.01	1869.40	28.03 ± 0.82	38.95 ± 0.35
1-Dodecanol	186.20	19.26	1966.08	9.24 ± 0.74	9.10 ± 0.68
Ester					
Methyl butyrate	102.07	3.79	978.00	37.91 ± 0.27	8.19 ± 0.97
Ethyl butyrate	116.08	4.60	1031.81	51.60 ± 0.89	607.84 ± 0.47
Butanoic acid, 2-methylpropyl ester	144.12	6.64	1150.09	ND	26.88 ± 0.30
Ethyl hexanoate	144.12	8.03	1227.60	4.25 ± 0.51	168.82 ± 0.63
Butanoic acid, 3-methylbutyl ester	158.13	8.62	1260.50	3.29 ± 0.58	50.87 ± 0.07
Ethyl octanoate	172.15	11.64	1436.68	55.99 ± 0.21	182.17 ± 0.16
Ethyl decanoate	200.18	14.78	1638.23	23.60 ± 0.74	313.61 ± 0.11
Ethyl dodecanoate	228.21	17.67	1844.22	112.40 ± 0.14	107.41 ± 0.28
Methyl hexadecanoate	270.26	22.29	2217.59	39.96 ± 0.47	30.74 ± 0.20
Ethyl hexadecanoate	284.27	22.72	2255.91	42.95 ± 0.71	40.25 ± 0.79

**Table 3:** Odour descriptors of selected volatile compounds, relative odour activity value (OAV) and odour threshold in water identified in inoculated milk samples are shown. * Odour threshold values in water were adapted from Leffingwell & Associates’ website [28]. Odour descriptions of volatiles were adapted from [30,31].

Compound	Odour Description	Threshold in Water (ppb) *	OAVs	
			Control	Thermo-Tolerant
Carboxylic acid				
Acetic acid	Vinegar, pungent acidic	800	2.02	1.49
Propanoic acid	Vinegar, pungent, sour milk	20,000	>1	>1
Butanoic acid	Fruity, dairy, cheesy	240	19.47	14.69
Hexanoic acid	Fatty, cheesy	3000	3.07	1.68
Octanoic acid	Fatty, cheesy	3000	1.41	>1
Nonanoic acid	Fatty, green	3000	>1	>1
Dodecanoic acid	Fatty, coconut, bay oil	10,000	>1	>1
Aldehyde				
Pentanal	Berry, nutty	1500	>1	>1
Heptanal	Green, sweet	3	50.87	8.88
4-Heptenal, (Z)-	Cream and butter	0.8–10	15.46	2.48
Nonanal	Fatty, citrus, green	1	57.98	33.29
2-Nonenal, (E)-	Fatty, green, mushroom	0.08–0.1	632.25	156.25
Ketone				
2-Butanone	Varnish-like, sweet, fruity	50,000	>1	>1
2-Pentanone	Fruity, acetone	70,000	>1	>1
2-Heptanone	Banana-like, fruity	140–3000	>1	14.61
2-Nonanone	Fruity, cheesy, buttery	5–200	19.65	376.31
3-Octen-2-one	Mushroom, fruity	28	>1	ND
2-Undecanone	Floral, rose-like, herbaceous	14.17	3.87	15.71
Alcohol				
2-Butanol	wine	500	>1	>1
1-Propanol	Alcoholic, pungent	9000	>1	>1
1-Butanol	Fruity, alcoholic	500	>1	>1
1-Pentanol	Alcoholic, iodoform-like	4000	>1	>1
2-Heptanol	Earthy oily	3	ND	139.98
1-Octen-3-ol	Mushroom-like	1	45.45	19.08
1-Heptanol	Earthy, oily	3	39.17	19.43
1-Octanol	pungent	110–130	>1	1.44
Ester				
Methyl butyrate	Fruity, apple, pineapple	60–76	>1	>1
Ethyl butyrate	Pineapple-like	1	51.60	607.84
Ethyl hexanoate	Fruity, apple, banana	1	4.25	168.82
Ethyl hexadecanoate	Fruity, creamy, waxy	>2000	>1	>1
